# Phospholipase A2 group IIA correlates with circulating high-density lipoprotein cholesterol and modulates cholesterol efflux possibly through regulation of PPAR-γ/LXR-α/ABCA1 in macrophages

**DOI:** 10.1186/s12967-021-03151-3

**Published:** 2021-11-27

**Authors:** Ling Liang, Qiang Xie, Changqing Sun, Yuanhui Wu, Wei Zhang, Weihua Li

**Affiliations:** 1 Department of Cardiology, Xiamen Key Laboratory of Cardiac Electrophysiology, Xiamen Institute of Cardiovascular Diseases, The First Affiliated Hospital of Xiamen University, School of Medicine, Xiamen University, Xiamen, 361003 China; 2 Department of Cardiology, The Third Clinical Medical College, Fujian Medical University, Fuzhou, 350122 China; 3 Department of Rheumatology and Clinical Immunology, The First Affiliated Hospital of Xiamen University, School of Medicine, Xiamen University, Xiamen, 361003 China

**Keywords:** Group IIA secretory phospholipase A2, High-density lipoprotein cholesterol, ATP-binding cassette A1, Cholesterol efflux, Peroxisome proliferator-activated receptor

## Abstract

**Background:**

Secretory phospholipase A2 group IIA (sPLA2-IIA) is an independent risk factor for cardiovascular disease, but its role on high-density lipoprotein cholesterol (HDL-C) level has not been clarified. The aim of the present study was to explore the association between circulating sPLA2-IIA and HDL-C, and to evaluate if sPLA2-IIA enhances cholesterol efflux capacity through regulation of peroxisome proliferator-activated receptor γ (PPAR-γ), liver X receptor α (LXR-α), and ATP-binding cassette A1 (ABCA1).

**Methods:**

131 patients with coronary artery disease were enrolled. The plasma level of sPLA2-IIA was tested with enzyme-linked immunosorbent assay kit, and serum lipids were assessed by biochemical analyzer. Human monocyte-macrophage cell line THP-1 was co-incubated with sPLA2-IIA in the presence/absence of selective PPAR-γ antagonist GW9662 in vitro. Real-time PCR and Western-blot were employed to measure the mRNA and protein expressions of PPAR-γ, LXR-α, and ABCA1, respectively. The cholesterol efflux was evaluated by using an assay kit.

**Results:**

In subjects, circulating level of sPLA2-IIA was positively related with that of HDL-C (r = 0.196, *p* = 0.024). The plasma level of sPLA2-IIA was significantly higher in the high HDL-C (≥ 1.04 mmol/L) group (7477.828 pg/mL) than that in low HDL-C (< 1.04 mmol/L) group (5836.92 pg/mL, *p* = 0.004). For each increase of 1 pg/μl in sPLA2-IIA level, the adjusted odds ratio for HDL-C ≥ 1.04 mmol/L was 1.143. Co-incubation of THP-1 cells with sPLA2-IIA resulted in increased expressions of PPAR-γ, LXR-α, and ABCA1, as well as enhanced cholesterol efflux capacity, that were all reversed by administration of GW9662.

**Conclusions:**

Circulating sPLA2-IIA was positively associated with HDL-C. PPAR-γ/LXR-α/ABCA1 might be responsible for sPLA2-IIA-regulated cholesterol efflux in macrophages.

**Supplementary Information:**

The online version contains supplementary material available at 10.1186/s12967-021-03151-3.

## Background

As a member of secretory phospholipase A2 (sPLA2) enzyme family, the group IIA (sPLA2-IIA) hydrolyses phospholipids from the membrane surface at the *sn*-2 position to liberate lysophospholipids and fatty acids [1]. The released free fatty acids triggers a cascade of cellular inflammatory reaction, acting as the initial molecule for the biosynthesis of eicosanoids and platelet-activating factor [2]. Eicosanoids, comprising prostaglandins, thromboxanes and leukotrienes, are closely associated with the pathogenesis of atherosclerosis [3]. Previous studies found not only high expression of sPLA2-IIA in the human plaque [4], and also the high activity of sPLA2-IIA predicting adverse events in acute coronary syndrome [5]. Moreover, sPLA2-IIA was increased in epicardial adipose tissue in patients with coronary artery disease [6]. Plasma level of sPLA2-IIA was considered to be related to functional characteristics of coronary stenosis [7].

However, the Mendelian randomized meta-analysis showed a null association between the sPLA2-IIA enzyme activity and major vascular events in general populations or in acute coronary syndrome patients [8]. Additionally, the phase III randomized clinical trial of secretory phospholipase A2 inhibitor (Varespladib) was terminated in advance due to the increased risk of myocardial infarction [9]. In transgenic mice over-expressing sPLA2-IIA, the serum concentrations of high-density lipoprotein cholesterol (HDL-C) and low-density lipoprotein cholesterol (LDL-C) were decreased [10] because the catabolic rate of cholesterol was faster than the control littermates [11], that was contradictory with the lower LDL-C levels the lower risk of major coronary events [12].

Given these inconsistencies, we hypothesized that sPLA2-IIA might exert some roles on cholesterol homeostasis. The present study was designed to explore the association between sPLA2-IIA and HDL-C in the development of coronary artery disease. By collecting blood samples from patients with coronary artery disease and by using the cell culture model of human monocyte-macrophage cells, we found that sPLA2-IIA exerted potent regulatory effect on cholesterol efflux through regulation of ABCA1. The possible involvement of PPAR-γ/ LXR-α signaling pathway was also evaluated.

## Methods

### Study population

Patients who were diagnosed with suspected coronary artery disease or confirmed coronary artery disease in the cardiovascular ward of the First Affiliated Hospital of Xiamen University from August 1 to December 31, 2018 were screened. The inclusion criteria were age > 18 years and coronary artery ≥ 50% stenosis by angiography. The exclusion criteria were acute myocardial infarction, heart failure, acute inflammation, malignancy and kidney dysfunction.

### Blood samples and biochemical analyses

Fasting blood samples were collected in the ethylenediaminetetraacetic acid vacutainers and the separation gel condensation vacutainers at the first morning of admission. The blood samples were immediately centrifuged at 3000 g at 4 °C for 10 min and the supernatants were frozen at − 80 °C for further testing. The lipoprotein levels (including HDL-C, LDL-C, total cholesterol (TC), and triglyceride (TG)) in the serums were analyzed in the Abbott Aeroset automatic biochemical analyzed (Chicago, IL, USA) based on the principle of spectrophotometry. Specific monoclonal antibody for human type IIA sPLA2 (Cayman Chemical Company, Ann Arbor, MI, USA) was used to detect the plasma sPLA2-IIA levels by the enzyme-linked immunosorbent assay (ELISA). The chemiluminescence method was applied to measure the high-sensitivity cardiac troponin I plasma levels.

### Cells culture conditions

Human monocyte-macrophage cell line (THP-1) was purchased from the Shanghai Institutes for Biological Sciences (Shanghai, China). Cells (1 $\times$ 10^6^ cells per well in one 6-well plate) were cultured and maintained in RPMI 1640 medium (Shanghai Institutes for Biological Sciences, Shanghai, China) containing 10% fetal bovine serum (Hyclone, Logan, Utah, USA) at 37 °C in a humidified atmosphere of 5% CO_2_. The medium was changed every 48–72 h. THP-1 were differentiated into macrophages by the addition of 160 nmol/L phorbol 12-myristate 13-acetate (PMA, Solarbio, Beijing, China) for 48 h.

THP-1 cells were exposed to sPLA2-IIA (0, 150 ng/ml, 300 ng/ml, 600 ng/ml) and different concentration of GW9662 (16 M$\mu$) for 24 h. GW9662 and sPLA2-IIA were purchased from MedChemExpress (MCE) company and R&D company in USA. In this study, control, sPLA2-IIA and sPLA2-IIA + GW9662 macrophages represented THP-1 cells that were treated with null, sPLA2-IIA and sPLA2-IIA + GW9662.

### Quantitative real-time PCR analysis of mRNA expression

Total RNA was extracted step by step using the RNeasy Mini Kit (Qiagen, German) in accordance with the manufacture’s protocol. Complementary DNA (cDNA) was synthesized by SuperScript IV kit (Invitrogen, Carlsbad, CA, USA) with Oligo (dT) primers according to the manufacture’s protocol. Real-time PCR was performed on Rotor Gene 6000 thermal cycler (Qiagen, German) with SYBER Ex Taq kit mixture (Takara, Japan) and primers detailed in Additional file 1: Table S1. The thermal cycling programs comprised ten minutes hot start at 95 °C, 40 cycles for 15 s at 95 °C, 40 cycles for 20 s at 60 °C prior to 40 cycles for 25 s at 72 °C, followed by 5 min at 72 °C The quality of amplified PCR products was evaluated by the dissociation curve analysis. CT values of different genes expression were calculated by using GAPDH as the reference gene and data were exported into an electronic spreadsheet.

### Western-blot

THP-1 cells were lysed by sonication in RIPA lysis buffer. The proteins content of the whole-cell lysates was determined by bicinchoninic acid assay (Thermo Scientific, MA, USA) before the proteins was separated on 10% SDS-PAGE gels. The semidry gels containing the separated proteins were transferred onto PVDF membranes. After being blocked in 5% (w/v) nonfat dry milk in Tris-buffered saline containing Tween-20 (TBST: 10 mM Tris, 150 mM NaCl, 0.1% Tween-20, pH 7.4) for one hour at room temperature, membranes were probed for PPAR-$\gamma$ (Abcam, final dilution 1:1000), LXR-$\alpha$ (Abcam; final dilution 1:1000), ABCA1 (Abcam; final dilution 1:1000), GAPDH (Abcam; final dilution 1:1000) overnight at 4 °C. The membranes were washes three time with TBST and goat mouse anti-rabbit IgG-HRP (Santa Cruz sc-2357; final dilution 1:5000) was used to probe the blots for one hour at room temperature. The proteins were visualized by enhanced chemiluminescence using ECL reagent (Thermo Supersignal West Pico, MA, USA). The films were scanned and analyzed with densitometry using ImageJ v1.49a (NIH).

### Cholesterol efflux assay

THP-1 cells were cultured in a 96 well plate using 100 μL media per well for two hours in a 37 °C incubator containing 5% CO_2_. When the cells were attached to the plates, the cell monolayer was washed with RPMI 1640 media (no serum added). 50 μL labeling reagent + 50 μL equilibration buffer mix from the cholesterol efflux assay (Abcam, U.K.) was added to the cells in every well. After being incubated overnight, the cells were washed gently using 200 μL RPMI media. The cholesterol acceptors were used to remove the rest labeling reagent. The cells were treated with 300 ng/ml sPLA2-IIA or 300 ng/ml sPLA2-IIA + 16 μM GW9662 in RPMI media for four hours in the incubator. The fluorescence of media and cells lysates were measured (Ex/Em = 482/515 nm). The percentage of cholesterol efflux was that the value obtained with fluorescence intensity of media was divided by the value obtained with fluorescence intensity of cell lysate plus fluorescence intensity of media × 100%.

### Statistics and endpoints

All data were recorded in the electronic spreadsheet. The continuous data were divided into normally distributed and non-normally distribution according to the Shapiro–Wilk test. To describe the normal distribution data, mean and standard deviation were used. The non-normal distribution data were reported as median and percentile intervals. The categorical data were expressed as numbers and percentages. To compare the continuous data between two independent groups, Student T test was used in the normal distribution data and Mann-Whiteney U-test was applied in the abnormal distribution. In the categorical comparison, cross-tabulated statistics were used and Chi-square test determined the level of significance. The Fisher exact test was applied if the expected frequency was less than five. The primary endpoint was to analyze the relationship between sPLA2-IIA and HDL-C. The scatter plot was drawn and curve fitting regression analysis were applied. If the data were normally distributed, Pearson correlation was used. Otherwise, Spearman correlation was applied for the non-normally distributed data. The secondary endpoint was to explore the potential mechanism of sPLA2-IIA regulating HDL-C biogenesis in vitro. A two-side *P* value less than 0.05 was considered to indicate statistically significant. Statistical analyses were carried out using the SPSS statistical software (version 24.0; SPSS Inc., Chicago, IL, USA).

## Results

### Demographics and baseline characteristics of patients

304 patients were screened initially. Totally, 131 patients were enrolled after exclusion of 28 cases with malignancy, 43 cases with acute myocardial infarction, 40 cases with inflammation, 11 cases with kidney dysfunction, 16 cases with heart failure and 35 cases without coronary stenosis. The included patients were divided into two groups: HDL-C < 1.04 mmol/L(low HDL-C group, n = 55), HDL-C ≥ 1.04 mmol/L(high HDL-C group, n = 76) based on the epidemiologic data [13]. The definition of unstable angina was in accordance with the guideline [14]. The baseline characteristics of all included patients were shown in Table 1. The dichotomous classification of HDL was significantly associated with sPLA2-IIA level (*p* = 0.004), LDL-C (*p* = 0.000), TC (*p* = 0.000), TG (*p* = 0.007). The sPLA2-IIA level, LDL-C, TC and TG were higher in the HDL-C ≥ 1.04 mmol/L group compared with those in the HDL-C < 1.04 mmol/L group.

**Table 1:** Baseline characteristics of included patients

	Low HDL-C (< 1.04 mmol/L)	High HDL-C (≥ 1.04 mmol/L)	*p* value
Male, n(%)	44 (80%)	50 (66%)	0.513
Age(year)	63.69 ± 11	63.03 ± 9.58	0.71
Hypertension, n(%)	33 (58%)	39 (51%)	0.419
Diabetes, n(%)	19 (35%)	18 (24%)	0.244
Smoking, n(%)	17 (31%)	22 (29%)	0.961
Gout, n(%)	2 (4%)	2 (3%)	0.56
LDL-C (mmol/L)	1.93 ± 0.79	2.67 ± 1.08	0.000
TC (mmol/L)	3.45 ± 0.87	4.85 ± 1.36	0.000
TG (mmol/L)	1.27 ± 0.48	1.79 ± 1.52	0.007
sPLA_2_ IIA (pg/mL)	5836.92 (3797.444–9198.456)	7477.828(5291.296–11,965.632)	0.004
Statin treatment, n(%)	32 (58%)	39 (51%)	0.548
hsCTNI level (ng/mL)	0.005 (0.002–0.011)	0.003 (0.001–0.009)	0.181
Unstable angina, n(%)	24 (44%)	21 (28%)	0.086
History of stent implantation, n(%)	10 (18%)	12 (19%)	0.901

*HDL-C* high-density lipoprotein cholesterol, *hsCTNI* hypersensitive cardiac troponin I, *LDL-C* low density lipoprotein-cholesterol, *sPLA2 IIA* secretory phospholipase A2 type IIA, *TC* total cholesterol, *TG* triglyceride

### The correlation and regression assessment of sPLA2-IIA and HDL-C

As the HDL-C levels were treated as the continuous data, the curve fitting regression analysis showed statistically significant relationship between sPLA2-IIA and HDL-C levels, by using the following models: linear, compound, power, growth, exponential, and logistic regressions (Table 2). Since the values of sPLA2-IIA level were non-normally distributed, the Spearman correlation of sPLA2-IIA and HDL-C was calculated and the r value was 0.196 (*p* = 0.024). Furthermore, the relationship between the levels of sPLA2-IIA and other types of lipoprotein cholesterol was analyzed by using Spearman correlation, but no significant correlations were found between sPLA2-IIA and LDL-C (*p* = 0.95), and sPLA2-IIA and LDL-C/HDL-C ratio (*p* = 0.127).

**Table 2:** Curve estimation in analyzing the relationship of HDL-C and sPLA2-IIA

Equation	Model summary					Parameter estimates			
	R Square	F	df1	df2	Sig	Constant	b1	b2	b3
Linear	0.035	4.621	1	129	0.033	1.051	6.08E−06		
Logarithm	0.028	3.739	1	129	0.055	0.601	0.057		
Inverse	0.007	0.86	1	129	0.355	1.125	− 111.321		
Quadratic	0.035	2.293	2	128	0.105	1.049	6.31E−06	− 6.29E−12	
Cubic	0.038	1.689	3	127	0.173	1.011	1.72E−05	− 6.98E−10	1.07E−14
Compound	0.036	4.765	1	129	0.031	1.027	1		
Power	0.03	3.972	1	129	0.048	0.673	0.053		
S	0.008	0.988	1	129	0.322	0.097	− 108.787		
Growth	0.036	4.765	1	129	0.031	0.027	5.63E−06		
Exponential	0.036	4.765	1	129	0.031	1.027	5.63E−06		
Logistic	0.036	4.765	1	129	0.031	0.973	1		

Independent variable: sPLA2-IIA; Dependent variable: HDL-C; *df* degree of freedom, *HDL-C* high-density lipoprotein cholesterol, *sPLA2-IIA* secretory phospholipase A2 type IIA

When the HDL-C levels were classified into dichotomous variables, sPLA2-IIA (pg/μL) predicted increased odds of HDL-C ≥ 1.04 mmol/L (OR = 1.143, 95% CI 1.024–1.274, *p* = 0.017) after adjustment for age, gender, hypertension, diabetes, smoke, gout, LDL-C, TC and TG in the stepwise multivariable logistic regression (Table 3). TC (mmol/L) also predicted increased odds of HDL-C ≥ 1.04 mmol/L (OR = 463.389, 95% CI 32.688 -6569.082, *p* = 0.000). In contrast, LDL-C (mmol/L) predicted decreased odds of HDL-C ≥ 1.04 mmol/L in the multivariable models (OR = 0.004, 95% CI 0–0.08, *p* = 0.000).

**Table 3:** Adjusted odds ratios of HDL-C ≥ 1.04 mmol/L

	Adjusted OR	95% CI	*p* value
Age	0.985	0.929–1.045	0.621
Male	3.856	0.907–16.397	0.068
Hypertension	1.825	0.616–5.411	0.278
Diabetes	1.537	0.467–5.064	0.48
Gout	4.714	0.227–97.91	0.316
Smoke	0.629	0.17–2.322	0.486
LDL-C (per mmol/L)	0.004	0–0.08	0.000
sPLA_2_ IIA (per pg/μL)	1.143	1.024–1.274	0.017
TC (per mmol/L)	463.389	32.688–6569.082	0.000
TG (per mmol/L)	0.759	0.3–1.919	0.56

*CI* confidence interval, *OR* odds ratio, *sPLA*_*2*_* IIA* secretory phospholipase A2 group IIA, *TC* total cholesterol, *TG* triglyceride

### sPLA2-IIA enhanced cholesterol efflux and ABCA1 expression in THP-1 cells in vitro

**Fig. 1:**
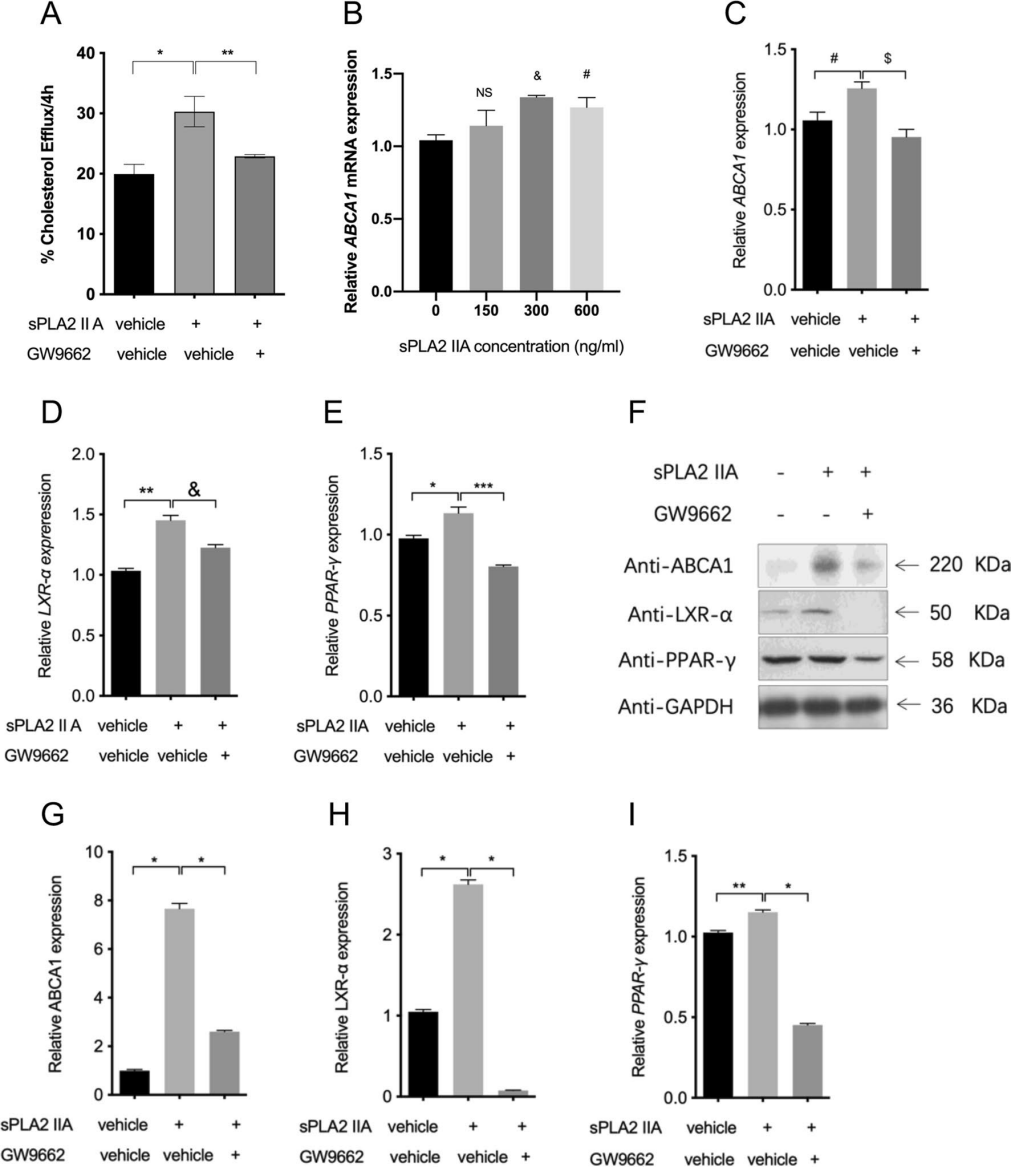
The effect of sPLA2-IIA on cholesterol efflux capacity and relative gene expression. **A** Cholesterol efflux capacity was significantly increased by sPLA2-IIA and GW9662 reversed the cholesterol efflux capacity. **B** ABCA1 mRNA expression was up-regulated in a dose-dependent manner. *p = 0.025, **p = 0.0424, NS = no significance, &*p* = 0.0002 versus control, #*p* = 0.0073 versus control, n = 3. Transcriptions levels of **C** ABCA1, **D** LXR-α, and **E** PPAR-γ in control, sPLA2-IIA and sPLA2-IIA (300 ng/ml) + GW9662 (16 μM) groups. #*p* = 0.0383, $*p* = 0.0081, **p = 0.006, &*p* = 0.0075, *p = 0.0195, ***p = 0.001, n = 3. Western blot results of PPAR-γ, LXR-α and ABCA1 protein expression in the control, sPLA2-IIA, sPLA2-IIA + GW9662 macrophages after co-incubation for 48 h. **F** A represented the blots of PPAR-γ, LXR-α and ABCA1 lanes. There were significantly greater amounts of **G** PPAR-γ, **H** LXR-α, and **I** ABCA1 in sPLA2-IIA macrophages compared with those in the control. Under the extra treatment of GW9662, the increased amounts of PPAR-γ, LXR-α, and ABCA1 were markedly reversed. *p = 0.000, **p = 0.0024, n = 3

To understand the role of sPLA2-IIA on cholesterol homeostasis, THP-1 cells were cultured with sPLA2-IIA or control (vehicle) for 4 h. The cholesterol efflux was significantly enhanced by sPLA2-IIA (Fig. 1A). The mRNA expression of ABCA1, one the most important mediator of cholesterol efflux, was up-regulated by sPLA2-IIA after co-incubation for 24 h (Fig. 1B).

### Changes in PPAR-γ, LXR-α and ABCA1 expression and cholesterol efflux

Since the mRNA expression of ABCA1 was up-regulated by sPLA2-IIA co-incubation, the involvement of the upstream mediator PPAR-γ/LXR-α signaling pathway was then evaluated by using PPAR-γ antagonist GW9662. After co-incubation with sPLA2-IIA for 24 h, the mRNA expression of ABCA1, LXR-α, and PPAR-γ was markedly elevated, and was abrogated by administration of GW9662 (Fig. 1C–E).

Western blot analysis showed that, the protein expression of PPAR-γ, LXR-α, and ABCA1 was increased in the sPLA2-IIA-treated cells as compared to that of control. GW9662 exerted inhibitory effects on the protein expression of PPAR-γ, LXR-α, and ABCA1 (Fig. 1F–I). Unsurprisingly, GW9662 reversed the increased cholesterol efflux capacity induced by sPLA2-IIA (Fig. 1A).

## Discussion

In the present study, a positive correlation between circulating sPLA2-IIA and HDL levels (r = 0.196, *p* = 0.024) was found in patient with coronary artery disease. As HDL level was categorized into dichotomous variables, plasma sPLA2-IIA level predicted 1.143 times odds ratio of HDL-C ≥ 1.04 mmol/L after adjustment for age, gender, hypertension, diabetes, smoke, gout, LDL-C, TC, and TG. Additionally, to the best of our knowledge, our in vitro data have for the first time demonstrated that the expression of PPAR-γ, LXR-α, and ABCA1 was upregulated after co-incubation of THP-1 cells with recombinant human sPLA2-IIA, paralleled with enhanced cholesterol efflux capacity. Selective PPAR-γ antagonist GW9662 not only downregulated the expression of PPAR-γ, LXR-α, and ABCA1, and also restrained the cholesterol efflux capacity of cells. These findings indicated the potent role of sPLA2-II2 on regulation of lipid homeostasis, possibly through regulation of ABCA1 in a PPAR-γ/LXR-α-dependent manner.

Following PLA2-IIA treatment, the structural change of lipoproteins caused the altered physiochemical and biological properties since the essential content of lipoproteins was phospholipids. The redistribution of free cholesterol from the core to the surface of lipoprotein particles, [15] and deeper sinking of apolipoproterins [16] after phospholipids hydrolysis, which increased excessive cholesterol accumulation in cells. However, the structure modification of HDL and LDL was not observed after treatment of the serum samples with high level of PLA2-IIA [17]. In mice model over-expressing sPLA2, the decreased concentration of HDL-C was due to the fast metabolism of HDL particles [11]. Besides, the lipoprotein levels remained unchanged in mice with macrophage-specific overexpression of sPLA2-IIA [18]. In the present study, we found that circulating sPLA2-IIA was positively correlated with HDL-C level in vivo, and boosted cholesterol efflux capacity in macrophages in vitro, suggesting that higher sPLA2-IIA might accelerate the process of reverse cholesterol transport.

The ATP-binding cassette (ABC) superfamily transporters utilize the energy of ATP hydrolysis to pump the substrate across the membrane against a concentration gradient, in order to protect organisms from xenobiotics [19]. Forty eight ABC genes in human are divided into seven subfamilies based on amino acid sequence similarities and phylogeny [20]. Among them, ABCA1 is required for transportation of cholesterol from peripheral cells, such as macrophages, into high-density lipoprotein particles [21]. This process is referred to as reverse cholesterol transport. ABCA1 played a pivotal role in the process of reverse cholesterol transport since the loss-function mutation of this gene caused severe early atherosclerosis (Tangier disease) [22].

Previous studies showed that, the expression of ABCA1 was tightly regulated by transcription factors, such as LXRs and PPAR-γ [23, 24]. The positive cross-talk between PPAR-γ and LXR-α activation on ABCA1 gene regulation was reported [25–27]. It has been confirmed that, in macrophages, PPAR-γ and LXR-α controlled the cholesterol removal through the modulation of ABCA1 [25]. Karina and colleagues revealed that, myotoxin III (an ophidian GIIA sPLA2) increased PPAR-γ and ABCA1 expression, and was involved in the lipid metabolism [28]. Consistently, our results indicated that sPLA2-IIA might increase the expression of ABCA1 through PPAR-γ/LXR-α pathway in THP-1 cells, and subsequently augment the cholesterol efflux capacity of cells. This might be beneficial for understanding the mechanisms underlying the clinical association between sPLA2-IIA and HDL-C.

Taken together, our findings could be potentially helpful for understanding the role of sPLA2-IIA on modification of lipid metabolism. Studies on the cardiovascular biomarkers broaden the current strategies in the diagnosis and treatment of disease. For instance, the clinical values of galectin 3 [29–31], heart-type fatty acid binding protein [32], and high sensitivity cardiac troponin I [33] have been evaluated and proven to be promising diagnostic/prognostic markers. The level of sPLA2-IIA per se has been demonstrated to be related to lipid modification and functional characteristics of coronary stenosis [7]. By investigating the underlying molecular mechanisms of sPLA2-IIA on alteration of HDL-C level, the translational significance could be developed to some extent.

There are some limitations: our findings of weak positive relationship between sPLA2-IIA and HDL-C might be underpowered and potentially confounded because of the relatively small sample size, although proper statistical methods were used to account for all the considered imbalances and risk factors. Additionally, the PPAR-γ/LXR-α/ABCA1 pathway could be responsible for sPLA2-IIA regulated cholesterol efflux, but further studies are required to elucidate the in-depth mechanisms.

## Conclusion

sPLA2-IIA levels were positively associated with HDL levels and predicted 1.143 times odds ratio of HDL-C ≥ 1.04 mmol/L in subjects. The upregulated ABCA1 expression by sPLA2-IIA, at least partly dependent on PPAR-γ/LXR-α pathway, could enhance cholesterol efflux capacity of THP-1 cells in vitro. Our study provided a new insight into the role of sPLA2-IIA in cholesterol metabolism.

## Supplementary Information

**Additional file 1: Table S1.** Primers sequences for RT-PCR.

## Data Availability

The datasets used and/or analyzed during the current study are available from the corresponding author on reasonable request.

## Abbreviations

sPLA2-IIA: Secretory phospholipase A2 group IIA
HDL-C: High-density lipoprotein cholesterol
PPAR-γ: Peroxisome proliferator-activated receptor γ
LXR-α: Liver X receptor α
ABCA1: ATP-binding cassette A1
ACS: Acute coronary syndrome
LDL-C: Low-density lipoprotein cholesterol
TC: Total cholesterol
TG: Triglyceride
ELISA: Enzyme-linked immunosorbent assay
THP-1: Human monocyte-macrophage cell line
