# Supplementary material for: Phospholipase A2 group IIA correlates with circulating high-density lipoprotein cholesterol and modulates cholesterol efflux possibly through regulation of PPAR-γ/LXR-α/ABCA1 in macrophages
Source: J Transl Med. 2021 Nov 27;19:484. doi: 10.1186/s12967-021-03151-3 (PMC8626914; doi:10.1186/s12967-021-03151-3)
Supplement: Supplementary file 1 — Additional file 1: Table S1. Primers sequences for RT-PCR. [file 12967_2021_3151_MOESM1_ESM.docx]

**Phospholipase A2 group IIA correlates with circulating high-density lipoprotein cholesterol and modulates cholesterol efflux possibly through regulation of PPAR-γ/LXR-α/ABCA1 in macrophages**

Ling Liang, Qiang Xie, Changqing Sun, Yuanhui Wu, Wei Zhang^*^ and Weihua Li^*^

**Additional file 1Tables**

Table S1. Primers sequences for RT-PCR

| Gene | Forward sequence (5’- -3’) | Reverse sequence (5’- -3’) |
| --- | --- | --- |
| Human ABCA1 | AACAGTTTGTGGCCCTTTTG | AGTTCCAGGCTGGGGTACTT |
| Human PPAR-γ | TGAATGTGAAGCCCATTGAA | CTGCAGTAGCTGCACGTGTT |
| Human LXR-α | AGTTTGCCTTGCTCATTGCT | AGACGCAGTGCAAACACTTG |
| Human GAPDH | TAGTACCGCTCGTGCAGGTTGA | CATGAGTCCTTCCACGATACC |

ABCA1: ATP-binding cassette A1; LXR-α: liver X receptor α; PPAR-γ: peroxisome proliferator-activated receptor γ.
